# The value of antinuclear antibody spectrum in diagnosis and disease activity assessment in early atypical systemic lupus erythematosus

**DOI:** 10.12669/pjms.42.7.14866

**Published:** 2026-07

**Authors:** Meichen Liu, Qing Liu, Meng Zhang, Dong Chen, Jianqiao Zhang

**Affiliations:** 1 Meichen Liu Department of Laboratory, Affiliated Hospital of Hebei University, Baoding 071000, Hebei, China; 2 Qing Liu Department of Laboratory, Affiliated Hospital of Hebei University, Baoding 071000, Hebei, China; 3 Meng Zhang Department of Laboratory, Affiliated Hospital of Hebei University, Baoding 071000, Hebei, China; 4 Dong Chen Department of Laboratory, The Sixth People’s Hospital of Hebei Province, Baoding 071000, Hebei, China.; 5 Jianqiao Zhang Department of Laboratory, Affiliated Hospital of Hebei University, Baoding 071000, Hebei, China

**Keywords:** Antinuclear Antibody Spectrum, Disease Activity, Early Atypical, Systemic Lupus Erythematosus

## Abstract

**Objectives:**

To explore the value of antinuclear antibody spectrum(ANAs) in diagnosis and disease activity assessment in early atypical systemic lupus erythematosus(SLE).

**Methodology:**

A retrospective analysis was conducted on the clinical data of 77 patients with early atypical SLE(the SLE group) from January 2023 to June 2025 in Affiliated Hospital of Hebei University, while the clinical data from 40 patients with other rheumatic diseases during the same period were selected as the non-SLE group. All 117 patients underwent serum ANAs detection using immunoblotting to compare the diagnostic value of the ANAs and combination detection for SLE. The 77 SLE patients were divided into the active(n= 43) and non-active disease(n= 34) groups following the SLE Disease Activity Index(SLEDAI), with multivariate logistic regression analysis adopted to identify risk factors affecting disease activity in SLE patients.

**Results:**

The positivity rates of ANA, anti-dsDNA, anti-Sm, anti-AunA, anti-SSA, anti-SSB, and anti-rRNP in the SLE group were significantly higher than those in the non-SLE group(*P*< 0.05), with no significant differences in the positivity rates of anti-SCL-70 and anti-Jo-1 between the two groups(*P*> 0.05). Meanwhile, the specificity of serum ANAs antibodies was high, with anti-Jo-1 showing the highest specificity at 100%. The multivariate logistic regression analysis indicated that positive anti-dsDNA and anti-AnuA were independent risk factors affecting disease activity in SLE patients(*P*< 0.05).

**Conclusion:**

ANAs is of significant value in diagnosis and disease activity assessment in early atypical SLE. More accurate diagnostic and clinical assessment references could be provided through the detection of various ANAs antibodies and their combinations, along with multivariate analysis.

## INTRODUCTION

Systemic lupus erythematosus (SLE) is a highly heterogeneous autoimmune disease characterized by complex and diverse clinical manifestations, which can affect multiple organs, including the skin, joints, kidneys, hematologic system, and central nervous system, thus severely threatening the quality of life and survival rates in patients.^1^ Due to the early atypical symptoms, SLE can easily be confused with other diseases, leading to delayed or missed diagnosis, ultimately resulting in delayed treatment.^2^ The antinuclear antibody spectrum (ANAs) serves as a core laboratory indicator for SLE diagnosis, among which the antinuclear antibodies (ANA) can be used for screening tests, which exhibit high sensitivity but low specificity, requiring the combination with other antibodies to enhance diagnostic accuracy.^3^ Dynamic monitoring of ANAs is of significant value for assessing disease activity and guiding treatment adjustments.^4^ However, limitations in the current application of ANAs detection remain. Therefore, this study is designed to explore the value of ANAs in diagnosis and disease activity assessment in early atypical SLE, thereby providing theoretical references for optimizing SLE diagnosis and treatment processes. The findings are reported as follows.

## METHODOLOGY

A retrospective analysis was conducted on the clinical data of 77 patients with early atypical SLE from January 2023 to June 2025 in Affiliated Hospital of Hebei University, who were designated as the SLE group. Additionally, clinical data from 40 patients with other rheumatic diseases during the same period were selected as the non-SLE group. All 117 patients underwent serum ANAs detection using immunoblotting to compare the diagnostic value of the ANAs and combination detection for SLE. The 77 SLE patients were divided into the active(n= 43) and non-active disease(n= 34) groups following the SLE Disease Activity Index(SLEDAI).

### Ethical approval:

The study was approved by the Institutional Ethics Committee of Affiliated Hospital of Hebei University (No.: HDFYLL-KY-2022-020; approval Date: December 30, 2022), and written informed consent was obtained from all participants.

### Inclusion criteria:

- Meeting the diagnostic criteria for SLE.^5^
- Age: ≥18 years.
- At the early stage of the disease, with a disease duration ≤2 years.
- Presenting with non-specific or mild symptoms, without well-defined and severe organ damage.
- Having complete clinical data and serological test results.

### Exclusion criteria:

- Having defined drug-induced lupus.
- Combined with other defined autoimmune disease overlap syndromes.
- Presence of defined severe organ damage.
- Treated with immunosuppressants or biological agents within the last three months.
- Pregnant or breastfeeding women.

The SLE group including 26 males and 51 females (age: 22 - 64 years, with an average age of (45.32±12.57) years; body mass index (BMI): 18 - 28 kg/m², with an average BMI of (22.96±2.96) kg/m²). The non-SLE group, which included 13 males and 27 females (age: 20 - 65 years, with an average age of (43.43±13.62) years; BMI: 18 - 25 kg/m², with an average BMI of (22.97±3.03) kg/m²). The disease types included rheumatoid arthritis (n = 23), connective tissue disease (n = 9), ankylosing spondylitis (n= 5), and systemic sclerosis (n= 3). There were no significant differences in the general data between the two groups (*P* > 0.05).

### Detection indicators and methods:

The detection of serum ANAs was based on the principle of indirect immunofluorescence. The experiment was performed using the ANA (IgG) detection kit (EUROIMMUN, Germany) along with biological slides (coated with human laryngeal carcinoma epithelial cells Hep-2 and monkey liver tissue), and the primary instrument was a Nikon 80i fluorescent microscope (Japan). The specific procedures were as follows: the serum to be tested was diluted at a ratio of 1:100 and added to the reaction area of the slide, incubated at room temperature for 30 minutes, and then thoroughly washed with phosphate-buffered saline containing Tween and rinsed for five minutes. Afterward, an isothiocyanate-fluorescein-labeled secondary antibody was added, with the washing and rinsing steps repeated after incubation under the same conditions for 30 min. Finally, the slides were sealed with glycerol and observed under a fluorescent microscope. The ANA fluorescence patterns were classified as per international consensus standards^6^, with a serum dilution titer ≥ 1:100 interpreted as positive.

The detection of specific antibodies in serum ANAs was based on the principle of immunoblotting. The experiment was conducted using the ANAs (IgG) detection kit (EUROIMMUN, Germany) and completed on EUROBlotMaster II, an automatic immunoblotting instrument. The detection covered the following nine types of autoantibodies: anti-double-stranded DNA (dsDNA), anti-Sm, anti-histone (AHA), anti-nucleosome (AnuA), anti-SSA, anti-SSB, anti-ribosomal P protein (rRNP), anti-Scl-70, and anti-Jo-1 antibodies. After the experiment was completed, the reacted membrane strips were air-dried, and images were obtained using a dedicated scanner, followed by automatic analysis with the accompanying EUROLineScan software. Afterward, the results were interpreted based on the grayscale values of the corresponding bands on the membrane strip: a grayscale value < 5 was deemed negative, 6-10 was considered a borderline value, and > 10 was considered positive.

### Disease activity assessment:

The disease activity of all SLE patients was assessed using the Systemic Lupus Erythematosus Disease Activity Index (SLEDAI).^7^ This scale provides a weighted score based on 24 clinical and laboratory indicators related to SLE that are present within 24 h (e.g., seizures, psychiatric symptoms, arthritis, proteinuria, low complement levels, elevated anti-dsDNA antibodies, etc.), with a total score of 0-105 points, effectively quantifying the overall activity intensity of the disease. In the meantime, an SLEDAI score of ≥ 10 points, widely used as a threshold for classifying disease activity in international studies, was employed. Accordingly, the 77 SLE patients were divided into two groups: those with an SLEDAI score < 10 points were classified as the stable disease group (n = 34), while those with an SLEDAI score ≥ 10 were classified as the active disease group (n = 43).

### Statistical analysis:

SPSS 22.0 was used for all statistical analyses. Normally distributed measurement data were expressed as(), and independent sample t-tests were used for inter-group analyses. Count data were expressed as n%, with inter-group comparisons performed using the chi-square (χ²) test. Additionally, risk factors affecting disease activity in SLE patients were identified through the multivariate logistic regression analysis. P < 0.05 was considered statistically significant.

## RESULTS

The positivity rates of ANA, anti-dsDNA, anti-Sm, anti-AHA, anti-AnuA, anti-SSA, anti-SSB, and anti-rRNP in the SLE group were higher than those in the non-SLE group (*P* < 0.05), with no significant differences in the positivity rates of anti-SCL-70 and anti-Jo-1 between the two groups (*P* > 0.05). Meanwhile, serum ANAs antibodies demonstrated high specificity, with the anti-Jo-1 antibody showing the highest specificity at 100%. Table-I and Table-II.

**Table-I:** Comparison of Positivity Rates of ANAs Antibodies between the Two Groups (n%).

Antibody Type	SLE Group (n = 77)	Non-SLE Group (n = 40)	χ^2^	P
ANA	72 (93.51)	30 (75.00)	8.067	0.005
Anti-dsDNA	55 (71.43)	8 (20.00)	28.016	<0.001
Anti-Sm	31 (40.26)	3 (7.50)	13.704	<0.001
Anti-AHA	53 (68.83)	9 (22.50)	22.684	<0.001
Anti-AnuA	45 (58.44)	6 (15.00)	20.204	<0.001
Anti-SSA	41 (53.25)	9 (22.50)	10.169	<0.001
Anti-SSB	23 (29.87)	1 (2.50)	12.095	<0.001
Anti-rRNP	14 (18.18)	1 (2.50)	5.792	0.016
Anti-SCL-70	5 (6.49)	1 (2.50)	0.863	0.353
Anti-Jo-1	2 (2.60)	0 (0.00)	1.057	0.304

**Table-II:** Comparison of Diagnostic Value of ANAs Antibodies between the Two Groups.

Antibody Type	Positive Patients (n)	Sensitivity (%)	Specificity (%)	Accuracy (%)
ANA	102	93.51	25.00	70.09
Anti-dsDNA	63	71.43	87.30	74.36
Anti-Sm	34	40.26	92.50	58.12
Anti-AHA	62	68.83	77.50	71.79
Anti-AnuA	51	58.44	85.00	67.52
Anti-SSA	50	53.25	77.50	61.54
Anti-SSB	24	29.87	97.50	52.99
Anti-rRNP	15	18.18	97.50	45.30
Anti-SCL-70	6	6.49	97.50	37.61
Anti-Jo-1	2	2.60	100.00	35.90

The positivity rates of anti-Sm + anti-dsDNA, anti-Sm + anti-AunA, anti-dsDNA+anti-AunA, anti-dsDNA + anti-rRNP, anti-AunA+anti-rRNP, anti-Sm + anti-dsDNA + anti-AnuA, and anti-dsDNA + anti-AnuA + anti-rRNP in the SLE group were higher than those in the non-SLE group (*P* < 0.05), with no significant differences noted in the positivity rates of anti-Sm + anti- rRNP, anti-Sm + anti-dsDNA + anti-rRNP, anti-Sm + anti-AnuA + anti-rRNP, anti-Sm + anti-dsDNA + anti-AunA+ anti-rRNP, and anti-Sm + anti-dsDNA+ anti-AunA + anti-rRNP + anti-AHA between the two groups (*P* > 0.05). In the meantime, all serum ANAs autoantibodies in combinations exhibited high specificity. Table-III and Table-IV.

**Table-III:** Comparison of Positivity Rates of Combined ANAs Autoantibodies between the Two Groups (n%).

Combination Mode	Combined Autoantibodies	SLE Group (n = 77)	Non-SLE Group (n = 40)	χ^2^	P
Dual Combination	Anti-Sm + anti-dsDNA	20 (25.97)	1 (2.50)	9.85	0.002
	Anti-Sm + anti-AunA	18 (23.38)	0 (0.00)	11.051	<0.001
	Anti-Sm+ anti-rRNP	6 (7.79)	0 (0.00)	3.285	0.070
	Anti-dsDNA +anti-AunA	34 (44.16)	3 (7.50)	16.358	<0.001
	Anti-dsDNA + anti-rRNP	11 (14.29)	0 (0.00)	6.307	0.012
	Anti-AunA + anti-rRNP	10 (12.99)	0 (0.00)	5.68	0.017
Triple Combination	Anti-Sm + anti-dsDNA + anti-AnuA	12 (15.58)	0 (0.00)	6.946	0.008
	Anti-Sm + anti-dsDNA + anti-rRNP	5 (6.49)	0 (0.00)	2.713	0.100
	Anti-Sm + anti-AnuA + anti-rRNP	4 (5.19)	0 (0.00)	2.151	0.142
	Anti-dsDNA + anti-AnuA+ anti-rRNP	8 (10.39)	0 (0.00)	4.461	0.035
Quadruple Combination	Anti-Sm + anti-dsDNA + anti-AunA + anti-rRNP	3 (3.90)	0 (0.00)	1.599	0.206
Quintuple Combination	Anti-Sm + anti-dsDNA + anti-AunA + anti-rRNP + anti-AHA	3 (3.90)	0 (0.00)	1.599	0.206

**Table-IV:** Comparison of diagnostic value of combined ANAs autoantibodies between the two groups.

Combination Mode	Combined Autoautibodies	Positive Patients (n)	Sensitivity (%)	Specificity (%)	Accuracy (%)
Dual Combination	Anti-Sm + anti-dsDNA	21	25.97	97.50	50.43
	Anti- Sm + anti-AunA	18	23.38	100.00	49.57
	Anti-Sm + anti-rRNP	6	7.79	100.00	39.32
	Anti-dsDNA + anti-AunA	37	44.16	92.50	60.68
	Anti-dsDNA + anti-rRNP	11	14.29	100.00	43.59
	Anti-AunA+ anti-rRNP	10	12.99	100.00	42.74
Triple Combination	Anti-Sm + anti-dsDNA + anti-AnuA	12	15.58	100.00	44.44
	Anti-Sm + anti-dsDNA + anti-rRNP	5	6.49	100.00	38.46
	Anti-Sm + anti-AnuA + anti-rRNP	4	5.19	100.00	37.61
	Anti-dsDNA + anti-AnuA + anti-rRNP	8	10.39	100.00	41.03
Quadruple Combination	Anti-Sm + anti-dsDNA + anti-AunA+ anti-rRNP	3	3.90	100.00	36.75
Quintuple Combination	Anti-Sm + anti-dsDNA+ anti-AunA + anti-rRNP + anti-AHA	3	3.90	100.00	36.75

The multivariate Logistic regression analysis was conducted using the disease activity of SLE patients as the dependent variable and ANAs antibodies as the independent variable with assigned values, suggesting that positive anti-dsDNA and anti-AnuA were independent risk factors for disease activity in SLE patients (*P* < 0.05). Table-V.

**Table-V:** Multivariate Logistic Regression Analysis of Disease Activity in SLE Patients.

Variable	B	SE	Wald χ^2^	P	OR	OR (95% CI)	
						Lower Limit	Upper Limit
ANA (+)	-0.213	1.010	0.045	0.833	0.808	0.112	5.852
Ani-dsDNA (+)	1.677	0.664	6.370	0.012	5.348	1.454	19.665
Anti-Sm (+)	-0.172	0.564	0.093	0.760	0.842	0.278	2.544
Anti-AHA (+)	0.096	0.620	0.024	0.877	1.101	0.327	3.709
Anti-AnuA (+)	1.159	0.579	4.004	0.045	3.187	1.024	9.919
Anti-SSA (+)	0.401	0.554	0.523	0.470	1.493	0.504	4.420
Anti-SSB (+)	1.077	0.622	3.001	0.083	2.937	0.868	9.937
Anti-rRNP (+)	0.788	0.719	1.201	0.273	2.199	0.537	9.003
Anti-SCL-70 (+)	1.564	1.287	1.477	0.224	4.779	0.383	59.563
Constant	-4.046	1.634	6.129	0.013	0.017		

## DISCUSSION

The findings of this study indicate that despite the high sensitivity of individual antinuclear antibody (ANA) as a diagnostic tool, its positivity rate can reach up to 75% in patients with non-SLE rheumatic diseases, significantly limiting its specificity for standalone diagnosis.^8^-^10^ In complex cases, a positive ANA result can easily lead to misdiagnosis.^11^ By contrast, anti-dsDNA and anti-Sm antibodies exhibit excellent diagnostic specificity, with anti-dsDNA demonstrating the optimal balance of sensitivity and specificity, making it a core indicator that bridges screening and definitive diagnosis.^12^ Meanwhile, anti-AnuA antibodies also exhibit high specificity and considerable sensitivity, receiving increasing attention for their diagnostic value. Pisetsky DS et al.^13^ similarly indicated that anti-AnuA emerges early in the disease and exhibits good complementarity with other specific antibodies. Conversely, the positivity rates of anti-Scl-70 and anti-Jo-1 antibodies are very low, without inter-group differences, further clarifying their primary use in the differential diagnosis of other specific connective tissue diseases.^14^ Combined detection is designed to optimize the balance between diagnostic sensitivity and specificity.^15^

This study found that combinations including highly specific antibodies (such as anti-Sm and anti-dsDNA) can achieve very high specificity and positive predictive value, but generally at the expense of sensitivity. For instance, the combination of anti-Sm and anti-AunA achieved a specificity of 100% but only a sensitivity of 23.38%, indicating that necessitating multiple positivity results may nearly confirm a diagnosis while risking misdiagnosing many patients. Conversely, the combination of anti-dsDNA and anti-AnuA, which includes two antibodies with relatively high positivity rates and associations with disease activity in SLE, achieved a better balance of sensitivity and specificity, demonstrating greater clinical application potential.^16^ In the diagnosis of early atypical SLE, instead of merely pursuing the theoretically highest specificity, a combination strategy should select antibodies with a good foundation of positivity rates in the target population to maximize detection rates while maintaining an acceptable risk of misdiagnosis.^17^

Additionally, Zeng Y et al.^18^ also proposed that incorporating preclinical probabilities and selecting combinations of indicators with moderate specificity but better sensitivity may provide greater identification efficiency for early atypical cases. Moreover, regarding the association between antibodies and disease activity, the multivariate logistic regression analysis identified positive anti-dsDNA and anti-AnuA as independent risk factors for disease activity. Anti-dsDNA antibodies serve as a serological marker of activity, particularly in lupus nephritis, with their mechanisms closely related to immune complex formation and complement activation.^19^ Anti-AnuA antibodies, as nucleosome-specific antibodies, have been widely confirmed to correlate with active renal damage and overall disease activity. Therefore, these findings extend the application of the antinuclear antibody spectrum from static diagnosis to dynamic assessment, emphasizing that monitoring serological activity markers should become an essential part in managing early SLE patients, providing alerts for disease flare-ups, and guiding treatment adjustments.^20^

### Limitations

It is a single-center, retrospective study with inherent risks of selection bias, as well as potential confounding factors that may not have been effectively controlled, all of which could influence the final outcomes. Given this, larger-scale, controlled trials with longer follow-up periods are expected in the future to further validate the specific mechanisms of ANAs while optimizing diagnostic protocols, so as to provide more scientific and reliable diagnostic references for patients with early atypical SLE.

## CONCLUSIONS

ANAs is of significant value in diagnosis and disease activity assessment in early atypical SLE, and more accurate diagnostic and disease assessment references could be provided through the detection of various ANAs antibodies and their combinations, along with multivariate analysis, making it beneficial for developing reasonable treatment regimens to improve patient outcomes.

## Author’s Contributions:

**ML:** Designed the study and conceived the survey, evaluated the results and revised the manuscript and are responsible and accountable for the accuracy or integrity of the work.

**QL** and **MZ:** Collected and analyzed clinical data. Critical Review.

**DC** and **JZ:** Did analysis and prepared the first draft of the manuscript.

All authors have read and approved the final manuscript.
